# Targeted immunotherapy: harnessing the immune system to battle multiple myeloma

**DOI:** 10.1038/s41420-024-01818-6

**Published:** 2024-01-27

**Authors:** Limei Xu, Caining Wen, Jiang Xia, Hao Zhang, Yujie Liang, Xiao Xu

**Affiliations:** 1 Department of Hematology, Affiliated Hospital of Jining Medical University, Jining Medical University, Jining, 272029 Shandong China; 2 Affiliated Hospital of Jining Medical University, Jining Medical University, Jining, 272029 Shandong China; 3 Department of Chemistry, the Chinese University of Hong Kong, Shatin, Hong Kong SAR, China; 4 College of Rehabilitation Medicine, Jining Medical University, Jining, 272029 Shandong China; 5 Shenzhen Second People’s Hospital, The First Affiliated Hospital of Shenzhen University, Shenzhen, 518035 China

**Keywords:** Cancer immunotherapy, Cancer immunotherapy

## Abstract

Multiple myeloma (MM) remains an incurable hematological malignancy disease characterized by the progressive dysfunction of the patient’s immune system. In this context, immunotherapy for MM has emerged as a prominent area of research in recent years. Various targeted immunotherapy strategies, such as monoclonal antibodies, antibody-drug conjugates, bispecific antibodies, chimeric antigen receptor T cells/natural killer (NK) cells, and checkpoint inhibitors have been developed for MM. This review aims to discuss promising experimental and clinical evidence as well as the mechanisms of action underlying these immunotherapies. Specifically, we will explore the design of exosome-based bispecific monoclonal antibodies that offer cell-free immunotherapy options. The treatment landscape for myeloma continues to evolve with the development of numerous emerging immunotherapies. Given their significant advantages in modulating the MM immune environment through immune-targeted therapy, these approaches provide novel perspectives in selecting cutting-edge treatments for MM.

## Facts

- The patients with Multiple myeloma present severely dysregulated immune systems.
- The utilization of immunotherapy drugs offers a highly effective therapeutic option for relapsed/refractory multiple myeloma patients.
- The application of immunotherapy demonstrates significant promise for newly diagnosed multiple myeloma patients.

## Open questions

- What are the potential applications for designing bispecific monoclonal antibodies based on exosomes?
- what are the effects of immunomodulatory drugs on the multiple myeloma cellular microenvironment?
- Can personalized combinations of immunotherapy drugs be provided to relapsed refractory patients to further enhance treatment outcomes?

## Introduction

Multiple myeloma (MM) is the second most prevalent hematological malignancy, characterized by abnormal proliferation of clonal plasma cells, resulting in hypercalcemia, renal insufficiency, anemia, bone disease, and other manifestations [1]. Despite significant advancements in MM treatment, it remains incurable. In the new therapeutic era, induction chemotherapy regimens based on proteasome inhibitors and/or immunomodulators as three or four-drug combinations combined with autologous stem cell transplantation have significantly prolonged the median survival time of MM patients. However, there are still cases where remission cannot be achieved or relapse occurs after remission [2–4].

**Fig. 1:**
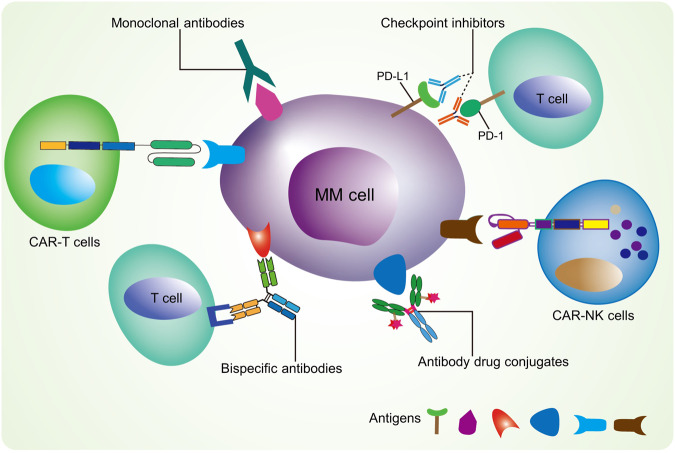
The targeted immunotherapeutic approaches for multiple myeloma. CAR chimeric antigen receptor, PD-L1 programmed death-ligand 1, PD-1 programmed cell death protein 1, TCR T cell receptor, MHC major histocompatibility complex, NK cell natural killer cell.

The pathogenesis of MM is complex, in which immune dysfunction plays a crucial role in disease recurrence and drug resistance [5]. It has been reported that myeloma cells escape immune detection, have insufficient antigen presentation cells, have T cell activation disorders, and other factors that can promote the survival of myeloma cells within the host [6]. With the continuous emergence of new drugs, the anti-tumor effect of the body’s autoimmune system can be stimulated by the action of plasma cell-specific antigens in patients with MM to achieve the purpose of targeting the killing of tumor cells. Therefore, the emergence of targeted immunotherapy has provided renewed hope for relapsed or refractory MM patients. Currently available, the targeted immunotherapies for MM mainly include chimeric antigen receptor T cell immunotherapy (CAR-T), CAR-NK cells, checkpoint-blocking antibodies, monoclonal antibodies, antibody-drug conjugates, and bispecific antibodies [7] (Fig. 1). This review aims to summarize the clinical application of these targeted immunotherapy strategies while introducing our novel exosome bispecific antibody-based immunotargeted therapy for MM. We hope this will inspire further advancements in targeted therapy.

## Cellular immunotherapy

### Chimeric antigen receptor T cells

**Fig. 2:**
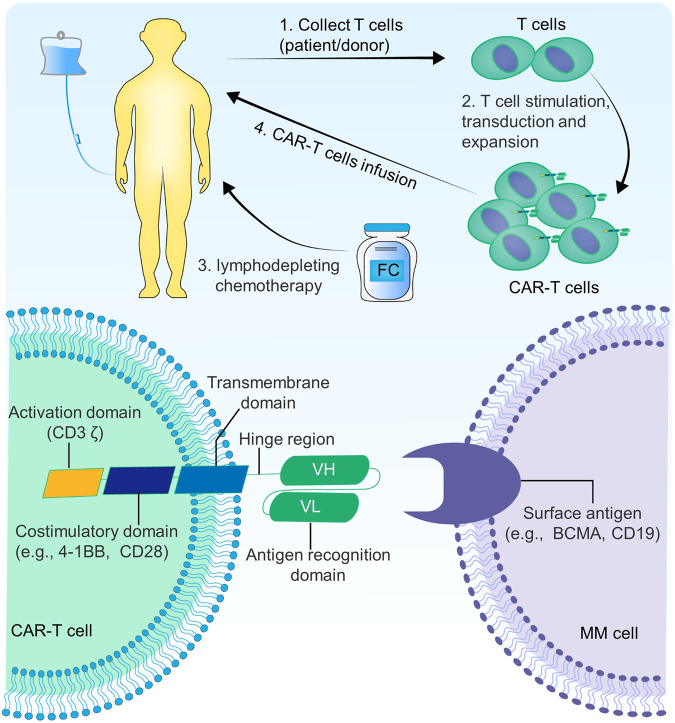
The chimeric antigen receptor (CAR) T cell treatment process for multiple myeloma and the structure of CAR-T cell. FC fludarabine and cyclophosphamide.

Chimeric antigen receptors (CARs) are transmembrane proteins consisting of an extracellular domain for antigen recognition, a hinge region, a transmembrane domain, and an intracellular signaling domain. The antigen recognition domain typically comprises single-chain antibodies (scFVs) that specifically target tumor antigens. The intracellular signaling domain includes the T cell activation domain (CD3 ζ) and costimulatory molecules necessary for T cell activation, usually including the costimulatory domains 4-1BB or CD28 [8–10]. The process of CAR-T cell treatment begins with the collection of T cells from either patient or healthy donors through leukapheresis. Subsequently, these T cells are stimulated, virally transduced, and proliferated to generate CAR-T cells by employing lentiviral or γ-retroviral vectors to transfer the CAR-encoding gene into the genome of T cells. Before infusing CAR-T cells, patients are initially treated with lymphodepleting chemotherapy, commonly using a regimen combining fludarabine and cyclophosphamide (FC). During the infusion of CAR-T cells, close attention should be paid to observing drug infusion reactions such as cytokine release syndrome (CRS) and neurotoxicity [11] (Fig. 2).

The antigens targeted by CAR-T cells should exhibit specific expression exclusively on the surface of tumor cells. B cell maturation antigen (BCMA) is a member of the tumor necrosis factor receptor superfamily, expressed in MM cells, while normal plasma cells and mature B cells exhibit low or no expression, making it the primary target for CAR-T cell therapy in MM patients [12]. The American Cancer Institute first launched a phase 1 clinical trial of anti-BCMA-CAR-T cell therapy for relapsed/refractory MM (R/RMM) (NCT02215967) demonstrated that BCMA-CAR-T cells are effective against R/RMM, but further optimization is needed to mitigate adverse effects [13, 14]. In a subsequent phase 1 clinical trial (NCT03090659), LCAR-B38M, a CAR-T cell targeting two different BCMA epitopes (VH1 and VH2), was reported in 57 patients who had received an average of three treatments with an ORR of 88%, a complete response (CR) rate of 68%, and a median progression-free survival (PFS) of 15 months. CRS occurred in 90% of subjects, primarily in grades 1 or 2(83%) [15]. CT103A is another BCMA-targeted CAR-T cell containing a fully human single-chain antibody, CD8α hinge, and transmembrane region, 4-1BB costimulatory and CD3ζ activation domains. In a phase I clinical trial (ChiCTR1800018137) of R/RMM being studied in China, good efficacy (ORR 100%; CR or strict CR rate 72.2%) was shown in 18R/RMM patients treated with CT103A. A subsequent phase I/II study (ChiCTR2000033946) included 71R/RMM patients to evaluate the efficacy and safety of CT103A. Notably, among these patients, 18.3% had previously received murine BCMA CAR-T therapy. The results were similarly encouraging (ORR 94.4%, MRD negative rate 92.8%). CRS occurred in 93.0% patients, of which only 2.8% were grade 3 [16, 17]. In addition to CT103A, BB2121 is another second-generation CAR incorporating an anti-BCMA single-chain variable fragment, a CD137 (4-1BB) costimulatory motif, and aCD3-zeta signaling domain. Both phase I (NCT02658929) [18] and phase II (NCT03361748) [19] clinical trials have shown favorable results in R/RMM, which were safe and manageable. The results of the phase II study showed that the ORR of 128R/RMM patients was 73% (≥CR rate 33%, MRD negative rate 26%), and the median PFS was 8.8 months. Based on these promising results, BB2121 has been approved by the FDA in 2020.

Other targets of CRA-T cell therapy mainly include CD19, CD38, and other antigens. A clinical trial (NCT02135406) reported that CAR-T targeting CD19 antigen combined with autologous hematopoietic stem cell transplantation in treating 10 patients with R/RMM, the ORR was 80% [20]. In addition, some studies have combined infusion of CD19 and BCMA-specific CAR T cells to treat R/RMM or high-risk MM with ORR ranging from 90 to 100% [21–23]. Besides, a study (ChiCTR1800018143) had reported that the results of a bispecific CAR-T cell therapy targeting BCMA and CD38 for R/RMM showed that after a median follow-up period of 9.0 months, 12 patients (52%) achieved a stringent complete response, and extramedullary plasmacytoma completely resolved in 56% of the patients [24]. Currently, there are some CAR-T cell therapy studies targeting other targets (such as CD138, SLAMF7/CS1, kappa light chain, etc.), but few studies have reported [25]. Table 1 summarizes the significant clinical trials of CAR-T therapy for MM.

**Table 1:** Summary of important clinical trials of CAR-T cells in multiple myeloma.

Agents	Study details	Patients	Regimens	Outcomes	Adverse events	Refs
anti-BCMA CAR-T cells	NCT02215967 Phase I	R/RMM at least 3 prior lines *n* = 24	LD chemotherapy followed by 0.3–9 × 10^6^ CAR-T cells/kg	ORR: 81% at 9 × 10^6^ CAR-T cells/kg; median EFS: 31 weeks	Any-grade CRS 94%, ≥grade 3 CRS 38%; ≥grade 3 NTX 19% at 9 × 106 CAR-T cells/kg	[13, 14]
	LCAR-B38M NCT03090659 Phase I	R/RMM at least 1 prior lines *n* = 57	LD chemotherapy followed by 0.07–2.1 × 10^6^ cells/kg CAR-T cells	ORR: 88%; median PFS 15 months	Any-grade CRS 90%, ≥grade 3 CRS 7%; any-grade NTX 1.7% (grade 1)	[15]
	CT103A ChiCTR1800018137 Phase I	R/RMM at least 3 prior lines *n* = 18	LD chemotherapy followed by 1–6 × 10^6^ CAR-T cells/kg	ORR: 100%; 1year PFS: 58.3%	Any-grade CRS 94%, ≥grade 3 CRS 22%; any-grade NTX 0%	[16]
	CT103A ChiCTR2000033946 Phase I/II	R/RMM at least 3 prior lines *n* = 71	LD chemotherapy followed by 1 × 10^6^ CAR-T cells/kg	ORR: 94.4%	Any-grade CRS 93%, ≥grade 3 CRS 2.8%; any-grade NTX 1.4% (grade 2)	[17]
	BB212 NCT02658929 Phase I	R/RMM at least 3 prior lines *n* = 33	LD chemotherapy followed by 50–800 × 10^6^ CAR-T cells/kg	ORR: 85%; median PFS 11.8 months	Any-grade CRS 76%, ≥grade 3 CRS 6%; ≥grade 3 NTX 3%	[18]
	BB212 NCT03361748 Phase II	R/RMM at least 3 prior lines *n* = 128	LD chemotherapy followed by 150–450 × 10^6^ CAR-T cells/kg	ORR: 73%; median PFS 8.6 months	Any-grade CRS 84%, ≥grade 3 CRS 5%; ≥grade 3 NTX 3%	[19]
anti-CD19 CAR-T cells	CTL019 NCT02135406 Phase I	R/RMM at least 2 prior lines *n* = 10	ASCT followed by 50 × 10^6^ CAR-T cells/kg	ORR: 80%	Any-grade CRS 10%, ≥grade 3 CRS 0%; no NTX reported	[20]
anti-CD19 CAR-T cells combined with anti-BCMA CAR-T cells	NCT 03196414 Phase I/II	R/RMM at least 1 prior lines *n* = 10	LD chemotherapy followed by 1 × 10^7^ CD19- CAR-T cells/kg day0, 3–6.5 × 10^7^ BCMA-CAR-T cells/kg day 1,2	ORR: 90%	Any-grade CRS 100%, ≥grade 3 CRS 10%; no NTX reported	[21]
	ChiCTR-OIC-17011272 Phase II	R/RMM at least 5 prior lines *n* = 22	LD chemotherapy followed by 1.0 × 10^6^ CD19- CAR-T cells/kg day0, 1 × 10^6^ BCMA-CAR T cells/kg day 0	ORR: 95%	Any-grade CRS 90%, ≥grade 3 CRS 5%; Any-grade NTX 9.5%	[22]
	SZ-MM-CART02 NCT 03455972 Phase I/II	NDMM High Risk *n* = 9	ASCT followed by 1 × 10^7^ CD19-CAR-T cells/kg day0, 5 × 10^7^ BCMA-CAR-T cells/kg day 1,2	ORR: 100%	Any-grade CRS 100%	[23]
anti-CD38 and anti-BCMA dual-target CAR-T cells	BM38 ChiCTR1800018143 Phase I	R/RMM at least 2 prior lines *n* = 23	LD chemotherapy followed by 0.5–4 × 10^6^ CAR-T cells/kg	CR: 87%; median PFS 17.2 months	Any-grade CRS 87%, ≥grade 3 CRS 35%; ≥grade 3 NTX 0%	[24]

*BCMA* B cell maturation antigen, *CAR* chimeric antigen receptor, *R/RMM* relapsed/refractory multiple myeloma, *LD* lymphodepleting, *ORR* overall response rate, *EFS* event-free survival, *CRS* cytokine-release syndrome, *NTX* neurotoxicity, *PFS* progression-free survival.

CAR-T therapy improves the survival and prognosis of patients with MM. However, current research on CAR-T therapy primarily focuses on R/RMM patients who exhibit resistance to multiple mechanistic drugs, necessitating further exploration of its therapeutic potential in other MM subgroups. Moreover, producing autologous CAR-T cells is a time-consuming process that requires strict control over transportation, storage, and management [26]. Additionally, optimizing and exploring off-target effects in CAR-T therapy, as well as addressing the severe side effects caused by CRS, remains crucial [27]. To enhance the safety of CAR-T cell therapy, studies have suggested incorporating a suicide switch into the CAR structure to trigger it when adverse reactions occur; however, only approximately 10% of clinical trials have successfully implemented this safety feature [28].

### Chimeric antigen receptor NK cells

Car-modified NK cells use genetic engineering to add a chimeric antibody to NK cells that can recognize tumor cells and simultaneously activate NK cells to kill tumor cells. It is safer than CAR-T cell therapy because NK cells do not secrete inflammatory factors such as IL-1 and IL-6. Since NK cells do not need to be pre-sensitized and do not cause graft-versus-host disease, Car-modified NK cells have attracted much attention recently [29].

Allogeneic NK cells come from various sources, including peripheral blood, NK cell lines, umbilical cord blood, induced pluripotent stem cells, etc [30]. Currently, CAR-NK cell therapy products for the research and treatment of MM mostly use the NK92 cell line. The cell surface glycoprotein CD2 subset 1 (CS1) is a highly expressed protein on the surface of MM cells and is primarily involved in the adhesion and growth of MM cells [31]. CD138 is highly expressed in MM cells and is the primary diagnostic marker for MM [32]. In vitro studies have shown that CS1-CAR-NK92 cells and CD138-IFNα-CAR-NK92 cells designed with CS1 and CD138 targets, respectively, successfully inhibited the growth of MM cells and prolonged the survival of myeloma mice [33, 34]. CAR-NK cell therapy also has some limitations: although NK92 cell lines can be easily amplified, their activation and proliferation depend on IL-2 or IL-15. Systematic IL-2 administration in clinical application has serious side effects and can activate immunosuppressive signals. In addition, improvement of CAR structure and transfection methods needs to be further optimized [30].

## Checkpoint inhibitors

**Fig. 3:**
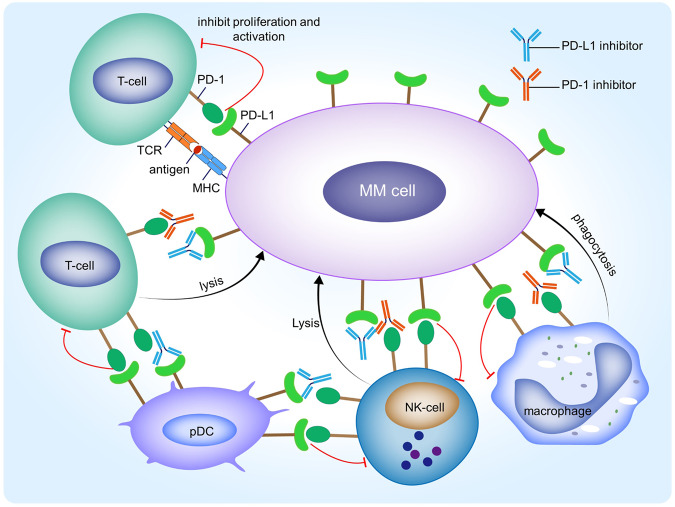
The mechanism underlying the therapeutic effects of checkpoint inhibitors in multiple myeloma. In patients with MM, the expression of PD-1 on various subsets of immune cells, including T cells, NK cells, and macrophages, is upregulated. The interaction between PD-1 on immune cells and PD-L1 on MM cells leads to the inhibition of immune cell proliferation and activation, resulting in the formation of an immunosuppressive microenvironment. Moreover, plasmacytoid dendritic cells (pDCs) exhibit high levels of PD-L1 expression, which further hampers the immune killing function when combined with PD-1 on T or NK cells. However, the administration of either PD-1 or PD-L1 inhibitors can disrupt the binding between PD-1 and PD-L1 molecules and restore the cytotoxicity of immune cells against MM.

Immune checkpoints refer to receptors and ligands involved in programmed cell death. The dysfunction of immune surveillance cells plays a crucial role in the development and progression of MM. Inhibiting the interaction between immune checkpoint receptors and their ligands can enhance the aggressiveness of the host immune system against tumor cells. Studies have demonstrated that aberrant activation of the programmed cell death protein 1 (PD-1)/ programmed death-ligand 1 (PD-L1) pathway is significant in MM [35]. The PD-1/PD-L1 axis functions as a negative costimulatory pathway, with increased expression of PD-1 on T cells and PD-L1 on plasma cells observed in MM patients. The binding of PD-L1 to PD-1 inhibits T cell proliferation and activation, creating an immunosuppressive microenvironment that promotes migration and proliferation of myeloma cells. By blocking the combination of PD-1 and PD-L1, inhibitors restore T-cell killing function against myeloma cells [36]. Furthermore, studies have shown elevated expression of PD-1 on other immune cells, such as NK cells and macrophages. Blocking the PD-1/PD-Ll pathway enhances NK-cell-mediated effects against MM and macrophage phagocytosis, thereby reducing tumor growth [37, 38]. Additionally, a study has demonstrated that pDCs and MM cells exhibit high expression of PD-L1, suggesting a dual inhibition of immune function in PD-1-expressing T cells and NK cells. Moreover, using PD-L1 inhibitors can augment the cytolytic activity of T cells and NK cells against MM cells [39] (Fig. 3).

Nivolumab and Pembrolizumab are PD-1 inhibitors. A Phase Ib trial evaluated Nivolumab monotherapy for R/RMM with an ORR of only 4% [40]. Another phase 1b study (KEYNOTE-013, NCT01953692) evaluated the efficacy of pembrolizumab monotherapy in the treatment of R/RMM and showed that the best response was disease stabilization in the 30 R/RMM patients enrolled (*n* = 17; 56.7%), and eventually 80% of patients stopped treatment due to disease progression/clinical progression (*n* = 24) [41]. All the above studies showed that PD-1 inhibitor monotherapy had poor efficacy in treating R/RMM. Consequently, researchers further investigated the synergistic potential of combining PD-1 inhibitors with other therapeutic agents in MM.

KEYNOTE-023 is a Phase I clinical study (NCT02036502) designed to evaluate the efficacy and safety of pembrolizumab in combination with lenalidomide and low-dose dexamethasone (Rd) in R/RMM. A total of 62 patients were enrolled, with an overall response rate of 44%, median PFS of 7.2 months, and 3.2% (2/62) of patients discontinued treatment due to TRAEs [42]. Although this study showed an acceptable safety and efficacy of pembrolizumab in combination with lenalidomide and low-dose dexamethasone for R/RMM, the results were not reproduced in the subsequent phase III trial. KEYNOTE-183 (NCT02576977) is a phase 3 clinical study that evaluated the efficacy and safety of pembrolizumab plus pomalidomide and dexamethasone (Pd) for R/RMM patients. A total of 249 patients were enrolled and randomly assigned to the pembrolizumab +Pd group or the Pd group. However, the results of the study showed that the pembrolizumab +Pd group had a shorter median PFS (5.6 months vs. 8.4 months), a decrease in the 6-month OS rate (82% vs. 90%), and a significantly higher proportion of serious adverse events (AEs) (63% vs. 46%) than the Pd group [43]. Similarly, the EYNOTE-185 (NCT02579863) phase 3 clinical study evaluated the efficacy and safety of lenalidomide and dexamethasone (Rd) with or without pembrolizumab in newly diagnosed MM (NDMM) patients who were not eligible for transplantation. Patients were randomly divided into the pembrolizumab + Rd group (*n* = 151) and Rd group (*n* = 150). The results of the study showed that compared with the Rd group, the 6-month PFS rate of the pembrolizumab combination group was reduced (82% vs. 85%), and the incidence of serious AEs was significantly higher (54% vs. 39%) [44]. Since the risks of the pembrolizumab combination outweighed the benefits, On 3 July 2017, the FDA halted the above study (The ASCO Post, 2017).

In addition, the majority of clinical trials investigating other PD-L1 inhibitors, such as durvalumab and atezolizumab, have been either completely or partially halted. The efficacy and safety of combining durvalumab with daratumumab in patients with daratumumab-refractory relapsed/refractory MM patients were evaluated in a prospective phase 2 clinical study (NCT03000452). The results revealed that the incidence of serious AEs was 38.9%, but none of the 18 enrolled patients achieved a PR or higher. Inhibition of the PD-1/PD-L1 signaling pathway during daratumumab resistance proved insufficient to reverse daratumumab resistance [45]. Table 2 provides an overview of the significant clinical trials involving checkpoint inhibitors in MM.

**Table 2:** Summary of significant clinical trials investigating checkpoint inhibitors in multiple myeloma.

Agents	Study details	Patients	Regimens	Outcomes	Adverse events	Refs.
PD-1 inhibitors	KEYNOTE-013 NCT01953692 Phase I b	R/RMM median 4 prior lines *n* = 30	pembrolizumab 10 mg/kg q2w or 200 mg q3w	ORR: 0%; median PFS 2.7 months	Common ≥grade 3 AEs: anemia (13.3%), hypercalcemia (6.7%)	[41]
	KEYNOTE-023 NCT02036502 Phase I	R/RMM at least 2 prior lines *n* = 62	Pembrolizumab+ Rd	ORR: 44%; median PFS 7.2 months	Common ≥grade 3 AEs: neutropenia (27.4%), thrombocytopenia (16.1%)	[42]
	KEYNOTE-183 NCT02576977 Phase III	R/RMM at least 2 prior lines *n* = 249	Pembrolizumab+ Pd vs. Pd	ORR: 34% vs. 40%; Median PFS: 5.6 vs. 8.4 months	Serious AEs: 63% vs. 46%	[43]
	EYNOTE-185 NCT02579863 Phase III	NDMM transplant ineligible *n* = 301	Pembrolizumab+ Rd vs. Rd	ORR: 64% vs. 62%; 6-month PFS: 82% vs. 85%	Serious AEs: 54% vs. 39%	[44]
PD-L1 inhibitor	NCT03000452 Phase II	R/RMM daratumumab- refractory *n* = 18	Durvalumab+ daratumumab	ORR: 0%	Serious AEs: 38.9%	[45]

*R/RMM* relapsed/refractory multiple myeloma, *ORR* overall response rate, *PFS* progression-free survival, *AEs* adverse events, *Rd* lenalidomide+ dexamethasone, *Pd* pomalidomide+ dexamethasone.

However, no specific cause or distinct AEs associated with a higher risk of death were observed in phase III clinical trials comparing PD-1 monoantibody combination therapy to the control group [46]. Other studies exploring pomalidomide and dexamethasone combined with pembrolizumab for treating relapsed/refractory MM demonstrated persistent immune effects even after discontinuation of pembrolizumab [47]. Additionally, a study investigating lenalidomide and dexamethasone combined with pembrolizumab for post-transplant consolidation therapy in high-risk myeloma patients suggested that this combination offers potential long-term disease control as a short-term consolidation regimen [48]. Therefore, PD-1 /PD-L1 inhibitor combination therapy for MM remains a therapeutic option.

## Monoclonal antibodies

**Fig. 4:**
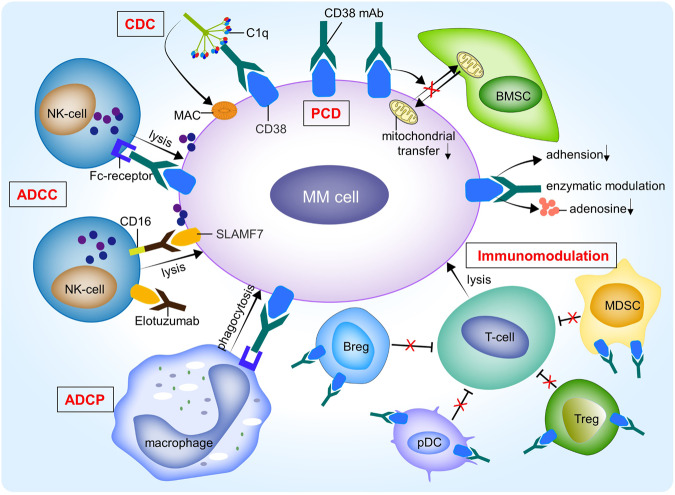
The mechanism of action underlying the therapeutic efficacy of monoclonal antibodies in multiple myeloma. Monoclonal antibodies have a variety of modes of action, mainly including antibody-mediated cross-linking induces programmed cell death (PCD) in MM cells; complement-dependent cytotoxicity (CDC) is initiated by the binding of C1q to the Fc tail of the antibody initiates the complement cascade, resulting in formation of the membrane attack complex (MAC); antibody-dependent cell-mediated cytotoxicity (ADCC) is the release of cytoplasmic granzymes and perforin by NK cells after being activated by antibodies, leading to apoptosis of MM cells; antibody-dependent cell-mediated phagocytosis (ADCP) is mainly the phagocytic destruction of MM cells mediated by macrophages; and immunoregulatory actions such as decreasing myeloid-derived suppressor cells (MDSCs), regulatory T cells (Tregs), regulatory B cells (Bregs), and pDCs, which is crucial for the activation of T cells. In addition, monoclonal antibodies can reduce mitochondrial transfer between bone marrow stromal cells (BMSCs) and MM cells, inhibit the function of adhesion molecules, modulate enzymatic activity, and reduce the production of adenosine to inhibit MM cells.

At present, the FDA has approved three monoclonal antibodies for the treatment of MM, including daratumumab (anti-CD38), elotuzumab (antibody targeting signaling lymphocytic activation molecule F7 (SLAMF7)), approved in 2015, and isatuximab (anti-CD38), approved in 2020. The mechanism of CD38 monoclonal antibodies (mAbs) treatment of MM is similar, such as complement-dependent cytotoxicity (CDC), antibody-dependent cell-mediated cytotoxicity (ADCC), and antibody-dependent cell-mediated phagocytosis (ADCP). In addition, CD38 mAbs can also induce programmed cell death, reduce mitochondrial transfer, inhibit adenosine production and adhesion molecule function, and regulate enzyme activity to cause MM cell death. Besides, CD38 mAbs can combine with immunomodulatory cells to eliminate the inhibitory effect of these cells on T effector cells, thereby activating T cells to kill MM cells [49, 50]. Elotuzumab works against MM mainly by directly activating NK cells and mediating ADCC through the CD16 pathway [51] (Fig. 4).

### Daratumumab

Daratumumab is the first CD38 monoclonal antibody for the treatment of MM. The results of clinical studies have confirmed that daratumumab alone or in combination can achieve good results in R/RMM. In Phase 1–2 clinical trials GEN501 (NCT00574288) and SIRIUS (NCT01985126), 16 mg/kg was determined to be the optimal dosage concentration for daratumumab [52, 53]. Subsequent phase III trials POLLUX [54–57] and CASTOR [58, 59] confirmed that daratumumab in combination with lenalidomide or bortezomib improved the remission rate of patients with R/RMM, including higher MRD negative rates and longer PFS and OS [60]. The recent open-label phase III trial, CANDOR (NCT03158688), enrolled 466 patients with R/RMM and randomly divided them into daratumumab + carfilzomib + dexamethasone (Dara-Kd) and Kd group. After 27.8 months of median follow-up, Dara-Kd prolonged PFS in the patients (median 28.6 vs 15.2 months; *P* < 0.0001). The incidence of AEs leading to discontinuation was similar in the D-Kd and Kd groups [61]. Overall, daratumumab-based combination therapy enables R/RMM patients to achieve deep remission and prolonged clinical outcomes in different subgroup analyses, including age, cytogenetics, etc [62, 63].

Currently, the utilization of daratumumab in combination with other chemotherapy regimens has witnessed a progressive rise in NDMM patients. For MM patients who are suitable for transplantation, the GRIFFIN (NCT02874742) phase II clinical trial included 207 patients, who were randomly divided into daratumumab, bortezomib, lenalidomide and dexamethasone (Dara-VRd) group and VRd group. The results confirmed that the complete remission rate, including deep remission, and the long-term survival prognosis of the Dara-VRd group were superior to that of the VRd group [64]. In a phase III clinical trial (CASSIOPEIA, NCT02541383) of 1085 patients with NDMM, the addition of daratumumab to a regimen of bortezomib, thalidomide, and dexamethasone (Dara-VTd) was associated with a higher rate of ≥CR rate and MRD negativity rate compared with standard triple therapy (VTd) after 100 days of transplantation. ≥CR rate was observed in 39% of patients in the Dara-treated group, with 64% achieving MRD negativity (10^−5^), while only 26% of patients with ≥CR, 44% being MRD negativity in the VTd group [65]. In addition, in terms of patient quality of life, Dara-VTd resulted in more significant pain reduction, less cognitive deterioration, and greater improvement in emotional function compared with the VTd group [66].

For newly diagnosed MM patients who are not suitable for transplantation, a series of phase III clinical studies have also been conducted; for example, the ALCYONE (NCT02195479) phase III clinical trial included 706 patients treated with nine cycles of daratumumab in combination with bortezomib, melphalan, and prednisone (Dara-VMP) or VMP. After chemotherapy, the remission rate and long-term survival were better in the Dara-VMP group than in the VMP group [67, 68]. In addition, the phase III clinical trial MAIA (NCT02252172) enrolled 737 NDMM patients and randomly divided them into daratumumab combined with lenalidomide and dexamethasone (Dara-Rd) group and Rd group. The ≥CR rates in the Dara-Rd and Rd groups were 47.6% and 24.9%, respectively (*p* < 0.001), and the MRD negative rates (10^−5^) were 24.2% and 7.3%, respectively (*P* < 0.001). At a median follow-up of 56.2 months, the median PFS was not reached in the daratumumab group versus 34.4 months in the control group (*p* < 0.0001) [69, 70]. The clinical benefit of Dara-Rd in transplant-ineligible NDMM patients enrolled in MAIA, regardless of frailty status [71]. At the same time, Dara-Rd also improves patients’ quality of life faster than Rd [72]. In terms of economic benefits, at the current price of daratumumab, first-line use of daratumumab is not cost-effective [73]. The 2022 V3 NCCN guidelines propose that for patients who are not suitable for hematopoietic stem cell transplantation, an appropriately reduced Dara-Rd regimen can be utilized, based on the aforementioned studies.

In general, for R/RMM patients and NDMM patients, daratumumab combined with standard therapy can be used to improve the curative effect, survival outcomes, and the patients’ quality of life. Furthermore, daratumumab-based combination therapy improved the depth of remission, that is, the rate of MRD-negative conversion and the duration of MRD-negative, which was also closely related to longer PFS [74, 75].

### Isatuximab

Isatuximab (SAR650984) is another humanized IgG1 monoclonal antibody targeting CD38-specific epitopes. The mechanism of action is similar to that of daratumumab. Still isatuximab can induce the internalization of CD38 in MM cells without releasing CD38 from MM cells, which is different from daratumumab [76]. There are also differences in the dose and frequency of administration. In comparison, the dose and frequency of Isatuxima are better than those of daratumumab [77].

Phase 1b clinical studies evaluated the favorable safety and efficacy of isatuximab in combination with lenalidomide and dexamethasone or isatuximab in combination with pomalidomide and dexamethasone in the treatment of patients with R/RMM [78, 79]. The phase III ICARIA study (NCT02990338) compared pomalidomide and dexamethasone in combination with Isatuximab in patients with bortezomib and/or lenalidomide-resistant R/RMM. A total of 307 patients were enrolled in this study, who were randomly divided into the Isatuximab/pomalidomide/dexamethasone group (Isa-Pd) and the Pd group. After a median follow-up of 11.6 months, the median PFS of the Isa-Pd group was better than that of the Pd group (11.5 vs. 6.5 months; *p* = 0.001). After a median follow-up of 35.3 months, the median OS of the Isa-Pd group was better than that of the Pd group (24.6 vs. 17.7 months; *p* = 0.001), and there was no significant difference in safety between the two groups [80, 81]. In addition, Isa-Pd improved ORR in patients with either lenalidomide-refractory, bortezomib-refractory, or double-refractory patients (59% vs. 31.4%; 60.2% vs. 32.2%; 58.6% vs. 29.9%; respectively) [82]. Subsequent subgroup analyses showed that the addition of Isa to Pd improved subpopulations with poor prognostic factors, including those with high-risk cytogenetics, those with renal impairment, those who are elderly, and those who are refractory to prior lines of treatment [83–85]. In addition, the IKEMA Phase III trial (NCT03275285) evaluated the efficacy of isatuximab + carfilzomib + dexamethasone (Isa-Kd) in R/RMM patients. A total of 302 patients were randomly divided into Isa-Kd and Kd groups. The results showed: Isa-Kd improved patients’ depth of response and median PFS (MRD negative rate 10^−5^: 30% vs. 16%, *P* = 0.0004; median PFS: not reached vs. 19.15 months, *p* = 0.0007; respectively). Serious AEs occurred similarly in both groups (59% vs. 57%) [86]. Subgroup analysis results showed that complete renal responses occurred more frequently with Isa-Kd (52.0%) versus Kd (30.8%) and were durable in 32.0% versus 7.7% of patients, respectively [87]. Based on these findings, the FDA has approved isatuximab in combination with pomalidomide, dexamethasone, and isatuximab in combination with carfilzomib, dexamethasone, respectively, for the treatment of R/RMM patients.

In addition, for newly diagnosed MM patients, a series of clinical studies have also been carried out. The GMMG-CONCEPT phase II clinical trial (NCT03104842) investigated the efficacy of four regimens of isatuximab, carfilzomib, lenalidomide, and dexamethasone (Isa-KRd) in high-risk (HR) newly diagnosed MM patients. The findings demonstrate encouraging rates of rapid and deep remissions in HR MM patients with Isa-KRd induction, with an ORR of 100% for the best induction response and an MRD-negative rate of 62.5%. After a median follow-up of 24.9 months, the median PFS was not reached, and the median 12-month PFS was 79.6%; the median 24-month PFS was 75.5% [88]. In addition, several ongoing clinical studies are currently being conducted to evaluate the combination of isatuximab with lenalidomide, bortezomib, and dexamethasone (GMMG-HD7), as well as the combination of isatuximab with carfilzomib, lenalidomide, and dexamethasone (NCT04430894) in NDMM patients eligible for transplantation. Furthermore, there are clinical studies assessing the efficacy and safety of isatuximab in combination with lenalidomide, bortezomib, and dexamethasone (Isa-VRd) for treating NDMM patients who are ineligible for transplantation (NCT02513186). The results from these studies are highly anticipated.

### Elotuzumab

Elotuzumab is a humanized IgG1 anti-SLAMF7 monoclonal antibody that elicits its effect via a dual mechanism of action: direct activation of NK cells and ADCC [51]. Although the efficacy of elotuzumab monotherapy in relapsed/refractory multiple myeloma (R/RMM) is not significant [89], its combination with other antineoplastic agents enhances patient response rates and improves clinical outcomes.

ELOQUENT-2 (NCT01239797) is a phase 3, randomized clinical trial that investigated the efficacy and safety of elotuzumab plus lenalidomide, dexamethasone (E-Rd) compared with Rd in R/RMM patients. The ORR in the elotuzumab group was 79% versus 66% in the control group (*P* < 0.001). Regarding security, infusion reactions occurred in 33 patients (10%) in the elotuzumab group and almost were grade 1 or 2 [90]. Subsequent follow-up data showed that, after a minimum follow-up of 70.6 months, the elotuzumab group also demonstrated a PFS advantage (median PFS: 19.4 versus 14.9 months; *P* < 0.001) and an OS advantage (median OS: 48.3 versus 39.6 months; *P* = 0.0408). The magnitude of OS benefit was most significant among patients with adverse prognostic factors, including older age, ISS stage III, IMWG high-risk disease, and 2–3 prior lines of therapy [91]. In addition, ELOQUENT-3 (NCT02654132) clinical studies have reported whether elotuzumab combined with pomalidomide + dexamethasone (EPd) is effective in treating R/RMM. The results showed that the ORR (53% vs. 26%) and PFS (median PFS: 10.3 versus 4.7 months; *P* = 0.008) of the elotuzumab group were better than those of the pomalidomide + dexamethasone (Pd) group, with no significant difference in adverse drug reactions [92]. Median OS was significantly improved with EPd (29.8 [22.9 to 45.7] months) versus Pd (17.4 [13.8 to 27.7] months), with a hazard ratio of 0.59 (95% CI, 0.37 to 0.93; *P* = 0.0217). OS benefit with EPd was observed in most patient subgroups [93]. Moreover, HRQoL was not impacted by the addition of elotuzumab to Pd [94]. The FDA has approved the use of elotuzumab in combination with lenalidomide/ dexamethasone or pomalidomide/dexamethasone for the treatment of R/RMM patients who have undergone 1–3 prior regimens, based on the studies mentioned above demonstrating the therapeutic benefits of elotuzumab-based drug combinations.

The SWOG-1211 (NCT01668719) study was conducted to further investigate the efficacy and safety of elotuzumab combined with lenalidomide, bortezomib, and dexamethasone (E-VRd) in newly diagnosed high-risk MM patients. However, the results showed no difference in median PFS (VRd 33.64 months vs. E-VRd 31.47 months, *p* = 0.45), and the addition of elotuzumab to VRd induction and maintenance did not improve patient outcomes [95].

### Other monoclonal antibodies

Currently, there are ongoing clinical developments for novel anti-CD38 monoclonal antibodies. For instance, phase I/II clinical studies have evaluated the efficacy and safety of MOR202 in combination with dexamethasone and/or lenalidomide and permadomide in R/RMM patients [96]. Furthermore, research is also underway to develop other targeted antibodies against malignant plasma cell surface molecules such as CD138 monoclonal antibody (indatuximab ravtansine), CD56 monoclonal antibody (lorvotuzumab mertansine), CD74 monoclonal antibody (Milatuzumab), CD40 monoclonal antibody (Dacetuzumab) and others. However, further studies are required to confirm the efficacy of these drugs’ efficacy and their combined [97–100].

Additionally, within the MM microenvironment consisting of stromal cells, endothelial cells, and osteoclasts, various growth factors, including interleukin-6 (IL-6), insulin-like growth factor 1(IGF1), vascular endothelial growth factor(VEGF) are secreted. These factors play crucial roles in activating intracellular signaling pathways that mediate MM cell growth, survival, migration, and drug resistance [101]. Therefore, the development of neutralizing growth factors or inhibiting growth-promoting receptor-related antibodies is underway. For instance, several studies have been conducted to assess the safety and efficacy of Siltuximab (anti-IL-6) in combination with conventional drugs for treating MM. Despite its good tolerability, the effectiveness of Siltuximab remains controversial [102–104]. Descamps et al. [105] discovered that AVE1642, a humanized IGF1 receptor monoclonal antibody, inhibited tumor growth and significantly enhanced bortezomib-induced apoptosis in CD45-negative tumor cell lines. However, the results of phase I studies of AVE1642 as a single agent and in combination with bortezomib in treating relapsed MM were not satisfactory [106]. Additionally, bevacizumab, a VEGF monoclonal antibody, can inhibit the activation of signaling cascades in MM cells and reduce their proliferation along with stromal cells [107]. Nevertheless, a randomized phase 2 clinical study evaluating the efficacy of bevacizumab combined with bortezomib in the treatment of R/RMM showed that it did not significantly improve the efficacy [108].

Monoclonal antibody immunotherapy has gradually emerged over recent years; FDA-approved examples include Dara monoclonal antibody, Isatuximab, and Elotuzumab. However, it remains crucial to further optimize drug performance and application methods while identifying specific targets for myeloma cells. Table 3 provides an overview of significant clinical trials involving monoclonal antibody therapy for MM.

**Table 3:** Summary of significant clinical trials evaluating the efficacy of monoclonal antibodies in multiple myeloma.

Agents	Study details	Patients	Regimens	Outcomes	Adverse events	Refs.
CD38 monoclonal antibodies	POLLUX NCT02076009 Phase III	R/RMM at least 1 prior line *n* = 569	Daratumumab+ Rd vs. Rd	ORR: 92.9% vs. 76.4%; median PFS: 44.5 vs. 17.5 months	Common ≥grade 3 AEs: neutropenia (55.5% vs. 41.6%), anemia (17.7% vs. 21.4%)	[54]
	CASTOR NCT02136134 Phase III	R/RMM at least 1 prior line *n* = 498	Daratumumab+ Vd vs. Vd	ORR: 83.8% vs. 63.2%; median PFS: 16.7 vs. 7.1 months	Common ≥grade 3 AEs: thrombocytopenia (45.7% vs. 32.9%), anemia (15.2% vs. 16.0%)	[58]
	CANDOR NCT03158688 Phase III	R/RMM at least 1 prior line *n* = 466	Daratumumab+ Kd vs. Kd	ORR: 84.3% vs. 74.7%; median PFS: 28.6 vs. 15.2 months	Common ≥grade 3 AEs: thrombocytopenia (25% vs. 16%), hypertension (21% vs. 15%)	[61]
	GRIFFIN NCT02874742 Phase II	NDMM transplant eligible *n* = 207	Daratumumab+ VRd vs. VRd	ORR: 99.0% vs. 91.8%; 1-year PFS: 95.8% vs. 89.8%	Common ≥grade 3 AEs: neutropenia (41.4% vs. 21.6%), lymphopenia (23.2% vs. 21.6%)	[64]
	CASSIOPEIA NCT02541383 Phase III	NDMM transplant eligible *n* = 1085	Daratumumab+ VTd vs. VTd	ORR: 92.6% vs. 89.9%; 18-month PFS: 93% vs. 85%	Common ≥grade 3 AEs: neutropenia (28% vs. 15%), lymphopenia (17% vs. 10%)	[65]
	ALCYONE NCT02195479 Phase III	NDMM transplant ineligible *n* = 706	Daratumumab+ VMP vs. VMP	ORR: 91% vs. 74%; 18-month PFS: 72% vs. 50%.	Common ≥grade 3 AEs: neutropenia (39.9% vs.38.7%), thrombocytopenia (34.4% vs. 37.6%)	[68]
	MAIA NCT02252172 Phase III	NDMM transplant ineligible *n* = 737	Daratumumab+ Rd vs. Rd	ORR: 93% vs. 81%; median PFS: not reached vs. 34.4 months	Common ≥grade 3 AEs: neutropenia (54% vs. 37%), pneumonia (19% vs. 11%)	[69]
	ICARIA NCT02990338 Phase III	R/RMM at least 2 prior lines *n* = 307	Isatuximab +Pd vs. Pd	ORR: 60% vs. 35%; median PFS: 11.5 vs. 6.5 months	Common any-grade AEs: infusion reactions (38% vs. 0%), pneumonia (20% vs. 17%)	[80]
	IKEMA NCT03275285 Phase III	R/RMM at least 1 prior lines *n* = 302	Isatuximab +Kd vs. Kd	ORR: 87% vs. 83%; median PFS: not reached vs. 19.15 months	Common ≥grade 3 AEs: respiratory infection (32% vs. 24%), thrombocytopenia (30% vs. 24%)	[86]
	GMMG-CONCEPT NCT03104842 Phase II	NDMM High-risk *n* = 50	Isatuximab+KRd	ORR: 100%; 24-month PFS: 75.5%	Common ≥grade 3 AEs: infectious (10%), cardiovascular disorders (10%)	[88]
SLAMF7 monoclonal antibodes	ELOQUENT-2 NCT01239797 Phase III	R/RMM at least 1 prior lines *n* = 646	Elotuzumab+ Rd vs. Rd	ORR: 79% vs. 66%; median PFS: 19.4 vs. 14.9 months	Common ≥grade 3 AEs: infections (35% vs. 27%), neutropenia (27% vs. 34%)	[90, 91]
	ELOQUENT-3 NCT02654132 Phase II	R/RMM at least 2 prior lines *n* = 117	Elotuzumab+ Pd vs. Pd	ORR: 53% vs. 26%; median PFS: 10.3 vs. 4.7 months	Common ≥grade 3 AEs: neutropenia (13% vs. 27%), anemia (10% vs.20%)	[92]
	SWOG S1211 NCT01668719 Phase II	NDMM High-risk *n* = 100	Elotuzumab+ VRd vs. VRd	ORR: 83% vs. 88%; median PFS: 31.47 vs. 33.64 months	Common ≥grade 3 AEs: infections (17% vs. 8%), sensory neuropathy (13% vs. 8%)	[95]

*R/RMM* relapsed/refractory multiple myeloma, *ORR* overall response rate, *PFS* progression-free survival, *AEs* adverse events, *Rd* lenalidomide + dexamethasone, *Vd* bortezomib + dexamethasone, *Kd* carfilzomib + dexamethasone, *VRd* bortezomib + lenalidomide + dexamethasone, *VTd* bortezomib + thalidomide + dexamethasone, *VMP* bortezomib + melphalan + and prednisone, *Pd* pomalidomide+ dexamethasone.

## Antibody-drug conjugates

**Fig. 5:**
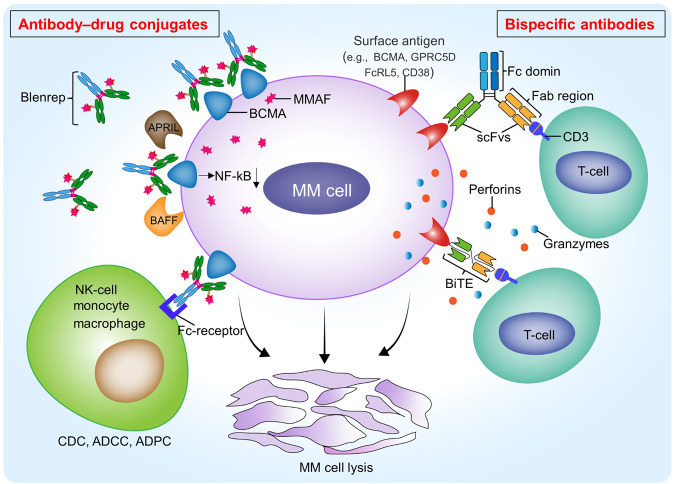
The mechanism of action underlying antibody-drug conjugates and bispecific antibodies in the treatment of multiple myeloma. Blenrep is an antibody-drug conjugate that specifically binds to BCMA on the MM cell surface, delivering the cytotoxic monomethyl auristatin F (MMAF) to kill MM cells. Blenrep could compete for the binding of B cell activation factor (BAFF) and/or a proliferation-inducing ligand (APRIL) to BCMA, thereby blocking the NF-κB signaling pathway, which is essential for MM cell growth. In addition, Blenrep could mediate the Fc-dependent effects consisting of CDC, ADCC and ADCP to kill MM cells. Bispecific antibodies (BsAbs), including bispecific T cell engagers (BiTE), could bind to both CD3 on T cells and MM cell surface antigens such as BCMA, GPRC5D, FCRL5 and CD38. The interaction leads to the activation and proliferation of T cells to release granzymes and perforins to kill MM cells. GPRC5D G-protein-coupled receptor class 5 member D, FcRL5 Fc receptor-like 5, scFvs single-chain variable fragments, Fab antigen-binding fragment, Fc crystallizable fragment.

Targeted monoclonal antibodies can also be used as carriers to deliver drugs to target cells. Among them, belantamab mafodotin (Blenrep, GSK-2857916) is a novel fucosylated anti-BCMA monoclonal antibody conjugated with the microtubule-disrupting agent monomethyl auristatin F (MMAF) via a protease-resistant linker. Blenrep targets and binds to BCMA on the surface of MM cells through an anti-BCMA monoclonal antibody, which is internalized by MM cells and releases MMAF, specifically blocks cell growth via G2/M arrest and induces caspase three dependent apoptosis in MM cells. At the same time, Blenrep inhibits B cell activation factor (BAFF)/a proliferation-inducing ligand (APRIL)-BCMA axis mediated NF-kB signaling by competitively binding to BCMA. Besides, Blenrep can also bind to effector cells (NK cells, monocytes, and macrophages) to promote ADCC and ADCP to kill MM cells [109, 110] (Fig. 5).

In the phase I study of the DREAMM-1 Study (NCT02064387), single-agent GSK2857916 had an ORR of 60% in R/RMM patients previously treated with alkylators, PIs, and immunomodulatory agents and refractory to the last line of therapy. After a median follow-up of 12.5 months, the median duration of response (DoR) was 14.3 months, and the median PFS was 12 months [111, 112]. The phase II study of DREAMM -2 (NCT03525678) enrolled 196 previously overtreated R/R MM patients to further evaluate the efficacy and safety of single-agent GSK2857916 in patients refractory to immunomodulators, PIs and refractory and/or intolerant to CD38-targeted monoclonal antibodies. Patients were randomly assigned to receive GSK2857916 at a dose of 2.5 mg/kg or 3.4 mg/kg once every 3 weeks. The ORR of GSK2857916 was 31% at 2.5 mg/kg and 34% at 3.4 mg/kg [113]. Subsequent analysis of GSK2857916 (3.4 mg/kg every 3 weeks) showed that after a median follow-up of 11.2 months, the median DoR was 9.0 months, the median PFS was 5.7 months, and the median OS was not reached. The most common grade 3/4 AEs were keratopathy (microcyst-like corneal epithelial changes, a pathological finding seen on eye examination [75%]); periodic ophthalmic evaluation is therefore required [114]. Based on these results, the FDA has approved GSK2857916 as a monotherapy for treating adult R/RMM patients who have received at least four prior therapies, including anti-CD38 monoclonal antibodies, PIs, and immunomodulators. Table 4 summarizes the significant clinical trials of GSK2857916 therapy for MM.

**Table 4:** Summary of important clinical trials investigating antibody-drug conjugates and bispecific antibodies in multiple myeloma.

Antibody-drug conjugate that binds to BCMA	DREAMM-1 NCT02064387 Phase I	R/RMM at least 5 prior lines *n* = 73	GSK2857916 0.03–4.60 mg/kg	ORR: 60% at 3.4 mg/kg; median PFS:12 months	Common ≥grade 3 AEs: corneal events (9%), thrombocytopenia (34%)	[111, 112]
	DREAMM-2 NCT03525678 Phase II	R/RMM at least 3 prior lines *n* = 196	GSK2857916 2.5 mg/kg or 3.4 mg/kg	ORR: 31% at 2.5 mg/kg, 34% at 3.4 mg/kg; median PFS: 5.7 months	Common ≥grade 3 AEs: keratopathy 27% at 2.5 mg/kg, 21% at 3.4 mg/kg	[113]
Anti-CD3/BCMA bispecific antibodies	NCT02514239 Phase I	R/RMM at least 2 prior lines *n* = 42	AMG420 0.2–800 μg/day	ORR: 31%(overall), 70% at 400 μg/d; median PFS: not reached	Common ≥grade 3 AEs: infections (19%), peripheral polyneuropathy (5%)	[119]
	NCT03933735 Phase I	R/RMM at least 3 prior lines *n* = 124	ABBV-383 0.025–120 mg/3 weeks	ORR: 68% at ≥40 mg	Common AEs: CRS (57%), neutropenia (37%), anemia (29%)	[120]
	NCT03145181 Phase I	R/RMM median 6 prior lines *n* = 165	Teclistamab 1.5 mg/kg/week	ORR: 63% median PFS: 11.3 months	Common ≥grade 3 AEs: CRS (0.6%), neutropenia (64.2%), anemia (37.0%)	[122]
	CC-93269-MM-001 NCT03486067 Phase I	R/RMM at least 3 prior lines *n* = 19	CC-93269 0.15–10 mg	ORR: 83.3% at ≥6 mg	Common ≥grade 3 AEs: neutropenia (52.6%), anemia (42.1%)	[123]
	MagnetisMM-1 NCT03269136 Phase I	R/RMM median 6 prior lines *n* = 58	Elranatamab 80-1000 μg/kg	ORR: 70% (overall), 83% at ≥1000 μg/kg	Common ≥grade 3 AEs: lymphopenia (64%), neutropenia (60%)	[124]
Anti-CD3/GPRC5D bispecific antibodies	NCT03399799 Phase I	R/RMM heavily pretreated *n* = 232	Talquetamab 0.5–180 μg/kg/week or biweekly or monthly	ORR: 70% at 405 g/kg, 64% at 800 g/kg weekly	Common AEs: CRS (77% and 80%), skin-related events (67% and 70%), and dysgeusia (63% and 57%)	[130]
Anti-CD3/FCRL5 bispecific antibodies	GO39775 NCT03275103 Phase I	R/RMM heavily pretreated *n* = 51	BFCR4350A step dose (0.05–3.6 mg), target dose (0.15–132 mg)	ORR: 51.7% at ≥3.6/20 mg	Common ≥grade 3 AEs: lymphocyte count decreased (11.8%), CRS (2%)	[132]

*BCMA* B cell maturation antigen, *R/RMM* relapsed/refractory multiple myeloma, *ORR* overall response rate, *PFS* progression-free survival, *AEs* adverse events, *CRS* cytokine-release syndrome, *IV* intravenous injection, *SC* subcutaneous injection.

In addition, other antibody-drug conjugates (ADCs) targeting BCMA are currently under development. The cytotoxic part of MEDI2228, for example, is tesirine, a DNA-binding pyrrole benzodiazepine (PBD) dimer that binds to antibodies via Linker, which can be cleaved by proteases [115]. The investigational drug AMG 224, currently undergoing phase I evaluation, also targets BCMA and is conjugated with the tubulin inhibitor mertansine (also known as DM1) [116].

## Bispecific antibodies

Bispecific antibodies (BsAbs) are antibodies capable of simultaneously targeting two antigens, typically the CD3 molecule of T cells and the tumor cell antigen. Recently, numerous BsAbs have been developed for MM treatment, with ideal tumor antigen targets including BCMA, G protein-coupled receptor 5D (GPRC5D), Fc receptor-like 5 (FCRL5), and CD38. BsAbs facilitate an immune synapse by recognizing and binding to surface antigens on T cells and MM cells, inducing T cell activation, proliferation, and subsequent tumor cell killing. There are various types of bispecific antibodies, mainly including the bispecific T cell engager (BiTE) containing two different scFvs connected by a flexible linker, and the IgG-like BsAbs consisting of fragment antigen-binding (Fab) domains and fragment-crystallizable (Fc) region [117]. While the Fc domain can mediate immune effects (such as ADCC, CDC, and ADCP), studies have shown that T-cell-directed BsAbs are designed to have an effector-silenced Fc region to prevent excessive cytokine secretion [118] (Fig. 5). Additionally, Table 4 summarizes significant clinical trials involving bispecific antibodies in MM.

### Anti-CD3/BCMA bispecific antibodies

As mentioned above, BCMA is expressed explicitly in MM cells and is an ideal target for treating MM. AMG 420 is a BiTE that targets BCMA on MM cells and CD3 on T cells. A dose-escalation trial was given to evaluate the efficacy and safety of AMG420 in R/RMM patients with up to 10 cycles of AMG 420 (4-week infusions/6-week cycles). The results showed that the maximum tolerated dose of AMG 420 was 400 μg/d, and the response rate was 70%, including 50% MRD-negative complete responses [119]. However, due to the short half-life of AMG420, continuous infusion is required to maintain blood concentration, which brings great inconvenience.

ABBV-383, a BCMA/CD3 T-cell engaging bispecific antibody with a silent human IgG4 Fc segment, effectively restricts nonspecific activation while exhibiting an extended half-life. ABBV-383 is in an ongoing phase I clinical trial in R/RMM patients who have received at least three prior line therapies (NCT03933735). Preliminary findings indicate that ABBV-383 demonstrated favorable tolerability and achieved an ORR of 68% at doses equal to or greater than 40 mg [120]. In addition, teclistamab, a bispecific IgG4 antibody, also demonstrated an extended half-life. A phase I study (NCT03145181) evaluating the efficacy and safety of teclistamab in R/RMM patients showed that teclistamab was well tolerated at a subcutaneous dose of 1500 ug/kg once per week, with no discontinuations due to treatment-emergent adverse events [121]. With a median follow-up of 14.1 months, the ORR was 63.0%, the MRD-negativity rate was 26.7%, and the median duration of PFS was 11.3 months (95% CI, 8.8–17.1). Based on these findings, an international, open-label phase 2 expansion study of teclistamab in patients with R/RMM is underway (NCT04557098) [122]. CC-93269 is an asymmetric 2-arm humanized IgG TCE that binds bivalently to BCMA and monovalently to CD3ε in a 2 + 1 format. Interim results of phase 1 multicenter clinical study (NCT03486067) of CC-93269 in patients with R/RMM: Among the 12 patients treated with ≥6 mg CC-93269 in cycle 1, the ORR was 83.3%, MRD-negativity rate was 75.0%. The most common adverse reactions are CRS (89.5%); however, the majority of these events were classified as grade 1-2 toxicity levels [123]. MagnetisMM-1 (NCT03269136) is a phase 1 study of elranatamab (PF-06863135), a humanized bispecific molecule that targets BCMA and CD3 for patients with R/RMM. The ORR was 70% (14/20) in 20 patients with an effective dose range of 215–1000 μg/kg once per week. CR/sCR rate was 30%, and negative MRD was induced in 100% of patients with evaluable MRD. At the highest dose of 1000 μg/kg, the ORR was 83% (*n* = 5/6). These results suggest that elranatamab, as a single agent, administered either q1w or q2w, had a manageable safety profile for patients with R/RMM. Other developments in combination with lenalidomide or pomadomide are still to be expected [124].

Several anti-BCMA /CD3 BsAbs are being evaluated in clinical trials. For example, animal experimental results showed that REGN5458 and AMG701 significantly inhibited the formation of MM tumors in murine xenogeneic models and showed potent combinatorial efficacy with programmed cell death protein one blockade [125, 126]. Notably, the combination of AMG 701 with lenalidomide induced sustained inhibition of MM cell growth in SCID mice reconstituted with human T cells. Therefore, clinical studies of AMG 701 as monotherapy in R/RMM patients and in combination with IMiDs to improve the outcome of MM patients are ongoing (NCT03287908) [127]. A phase 1 study of REGN5458 in heavily pretreated patients (NCT03761108) is ongoing. Although BCMA as an MM cell antigen has been used to develop various bispecific antibodies, BCMA antigen loss may occur during treatment, so the development of other antigens is still very important.

### Anti-CD3/GPRC5D bispecific antibodies

G-protein-coupled receptor class 5 member D (GPRC5D) is a 7-pass transmembrane protein. Studies have shown that GPRC5D is significantly expressed in malignant plasma cells and has limited expression in normal human tissue, making it an attractive novel antigen for MM cells to target. This study synthesized a GPRC5DxCD3 bispecific antibody (Jnj-64407564) that can recruit CD3+ T cells to GPRC5D+ MM cells and induce killing of GPRC5D+ cells. In vivo experiments in a mouse myeloma model showed that JNJ-64407564 recruited T cells and induced tumor regression [128].

Based on the results of previous studies, the efficacy and safety of talquetamab (JNJ-64407564) is currently being evaluated in a phase I clinical trial (NCT03399799) in R/RMM patients, 30% of whom had previously been treated with BCMA. The results showed that in 30 response-evaluable patients treated with the recommended phase 2 doses (RP2Ds 405 µ g/kg SC weekly), the ORR was 70%, and the 6-month DOR rate was 67% (95% CI: 41–84). Responses were durable and deepened over time. The most common AEs at 405 g/kg weekly dose were CRS (73%; 1 patient had grade 3 CRS), neutropenia (67%), and dysgeusia (60%). Overall, talquetamab had a tolerable safety profile at the RP2D dose, with no dose-limiting toxicity (DLT) observed [129]. At median follow-ups of 11.7 months, the percentage of patients with a response was 70% (95% CI, 51 to 85), and the median duration of response was 10.2 months. Further studies of talquetamab monotherapy (phase II, NCT04634552) and in combination with other therapies to treat patients with R/RMM are underway [130].

### Anti-CD3/FcRL5 bispecific antibodies

Fc receptor-like 5 (FcRL5/FcRH5/IRTA2/CD307) is a type I membrane protein with an Ig domain. Studies have shown that the expression rate of FcRH5 in myeloma cells is 100%, which is significantly higher than that in normal B cells. This study developed and preclinically validated an anti- FcRL5/CD3 TDB as an immunotherapy for MM. Anti-FcRL5/CD3 TDB (BFCR4350A) induced mild/moderate cytokine release immediately after administration in primates, effectively killing myeloma cells and marrow plasma cells, but the effect in mouse models was significantly limited [131].

GO39775 (NCT03275103) is an ongoing multicenter phase I clinical trial designed to evaluate the safety, activity, pharmacodynamics, and pharmacokinetics of BFCR4350A monotherapy in R/RMM patients. All enrolled patients were previously exposed to CAR-T cells, T-cell-engaging bispecific antibodies, and antibody-drug conjugates, including those targeting BCMA, for which no established therapy is available, appropriate, or tolerated. At the cut-off time, ORR was 51.7% (15/29), observed at dose levels of 3.6/20 mg and above. Notably, treatment responses were also observed in high-risk patients: those with HR cytogenetics (9/17), triple-class refractory disease (10/20), and prior exposure to anti-CD38 monoclonal antibodies (11/22), CAR-T cells (2/3), or antibody-drug conjugates (2/2). The most common treatment-related AE was CRS (74.5%), most of which were grade 1–2 (72.2%), and most occurred in the first course of treatment. These results indicate that BFCR4350A monotherapy shows promising efficacy with manageable toxicity in heavily pretreated R/R MM [132].

### Anti-CD3/CD38 bispecific antibodies

CD38 is a membrane protein universally expressed at high levels in MM cells. AMG 424, a novel humanized T cell-recruiting bispecific anti-CD3/CD38 antibody, has been described recently. In vivo, AMG 424 could inhibit tumor growth in bone marrow invasive mouse cancer models and the depletion of peripheral B cells in cynomolgus monkeys without triggering excessive cytokine release. However, the activity of AMG 424 against normal immune cells expressing CD38 is also presented [133]. Another study developed a new CD3/CD38 BiTE (BI38-3) consisting of two single-stranded variable fragments from mouse hybridomas. This study has shown that BI38-3 triggers T cell-mediated lysis of CD38+ MM cells both in vitro and in vivo. Bi38-3 does not impair the surface expression of CD38 and only triggers T-cell-mediated killing of cells expressing high levels of CD38 while showing no or limited toxicity to cells expressing intermediate levels of CD38 (e.g., hematopoietic progenitors, B, T, or NK cells). Therefore, compared with AMG424, BI38-3 can trigger the killing of MM cells more effectively. In addition, because BI38-3 recognizes a specific epitope on CD38 and does not have an Fc region, it is expected to be effective in patients who relapsed after daratumumab treatment [134]. However, the clinical trials of these two CD3/CD38 bispecific antibodies have not yet been investigated, and further research progress is anticipated.

## Our new findings on immunotherapy for multiple myeloma

Despite advances in bispecific antibodies, many patients do not respond to them or relapse afterward. Therefore, it is crucial to optimize the performance of bispecific antibodies continuously [135]. Exosomes are vesicle-like nanobodies secreted by many kinds of cells, which have the advantages of low immunogenicity, strong stability, and good biocompatibility [136]. Signals are transferred from exosomes to recipient cells by receptor-ligand interactions, direct membrane fusion, and endocytosis/ phagocytosis [137]. Exosomes are widely present in human blood, saliva, and other body fluids. They can stably deliver microRNAs, small interfering RNAs, and anticancer drugs to target cells, which has an extensive application prospect in drug delivery [138, 139]. Although natural exosomes are not targeted, they can be genetically engineered or chemically engineered to obtain targeting to recipient cells [140]. Numerous preclinical and clinical studies have shown that exosome-based therapeutics have good efficacy and safety in treating many human diseases [141, 142].

One study expresses anti-CD3 and anti-tumor cell associated epidermal growth factor receptor (EGFR) molecules on the surface of exosomes by genetic engineering, thereby generating a drug (aCD3-a EGFR SMART-Exos) that simultaneously targets T cells (CD3) and tumor cells (EGFR), activating cytotoxic T cells to attack tumor cells expressing EGFR. aCD3-a EGFR SMART-Exos exhibited highly potent and specific anti-tumor activity in vitro and in vivo [143]. Another study also synthesized SMART-Exos that could target T cells (CD3) and tumor cells (HER2). The results demonstrated that aCD3-aHER2 SMART-Exos exhibited enhanced binding affinity towards both CD3+ and HER2+ cell lines in vitro, mediating the interaction between T cells and HER2+ tumor cells, thereby activating cytotoxic T cells to target HER2-expressing cancer cells effectively. Mice treated with aCD3-aHER2 SMART-Exos displayed remarkable suppression of tumor growth without any observed liver or kidney damage. Furthermore, the study also compared aCD3-aHER2 SMART-Exos with bispecific aCD3-aHER2 antibodies, revealing that αCD3-αHER2 SMART-Exos exhibited superior potency in inducing specific cytotoxicity against HER2-positive cells and promoting T-cell activation [144].

**Fig. 6:**
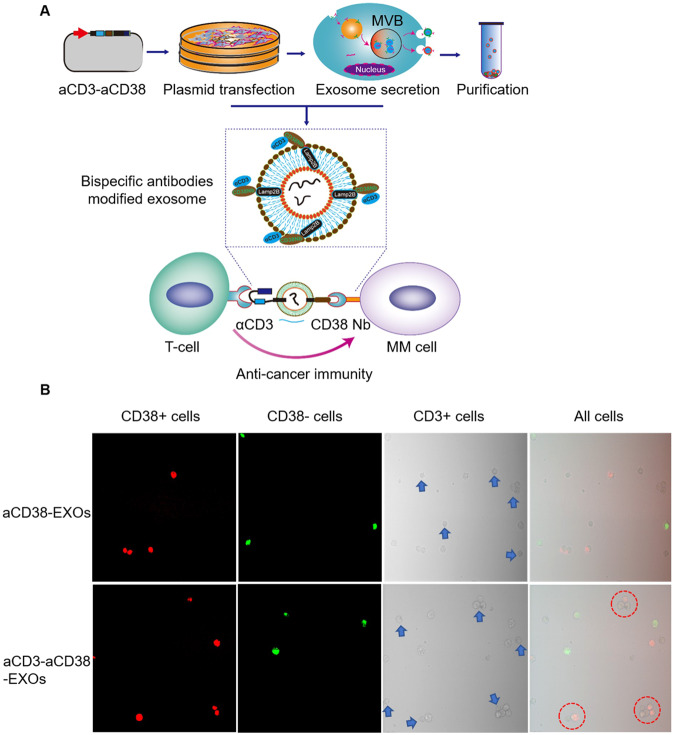
Bispecific antibody labeling of exosomes was used as synthetic organelles that bridge the T cell and CD38+ myeloma cell. **A** Construction process and action mode of bispecific antibodies modified exosomes (aCD3-aCD38-EXOs); **B** Compared with aCD38-EXOs, aCD3-aCD38-EXOs mediated the binding of T cells and MM cells to each other.

Since we have done much essential research on modifying targeted antigens and drug delivery on exosomes [145–147], we attempted to achieve its dual targeting to T cells and myeloma cells through exosome surface protein modification. A flexible (GGGGS)3-linker spliced CD3 scFvs gene sequence and CD38 nano antibody (Nb) gene sequence were linked to the Lamp2b gene to construct a double-targeted fusion expression plasmid. Subsequently, we transfected aCD3-aCD38 Nb-Lamp2b plasmid into Expi293F cells to obtain exosomes that could bind CD3 + T cells and CD38 + MM cells simultaneously. We next performed in vitro cell interaction experiments using CD3+ T cell line (Jurkat), CD38+ myeloma cell line (NCI-H929), and CD38-cell line (K562). We co-incubated red fluorescently labeled CD38+ cells, green fluorescently labeled CD38- cells, and CD3+ cells with aCD3-aCD38 -EXOs to observe the cell-cell interactions by confocal microscopy. Results showed that CD3+ Jurkat cells were significantly crosslinked with CD38 + NCI-H929 cells, but no cross-linking of CD3+ Jurkat cells and CD38- K562 cells. These data supported aCD3-aCD38 -EXOs induced cell-cell interactions (Fig. 6).

For the first time, we genetically engineered exosomes to target both CD3+ T cells and CD38+ MM cells, giving them the benefits of bispecific antibodies. In addition, taking advantage of the drug-loading advantages of exosomes, cytotoxic drugs can be loaded into bispecific antibody-targeted exosomes in vitro to exert both immune-killing and cytotoxic effects. This study design may provide a theoretical basis for developing a novel immunotherapy-coupled cytotoxic drug therapy.

## Conclusion

In recent years, the application of proteasome inhibitors and immunomodulatory agents in MM has significantly prolonged the survival of patients. However, due to the heterogeneity of MM, the disease is prone to recurrence and drug resistance. New immunotarget therapy has brought great promise for the treatment of MM. In particular, CAR-T and monoclonal antibody therapy have shown promising efficacy in inducing deep and durable remissions in both NDMM and R/RMM. In addition, BiTEs and antibody-drug conjugate treatment have also demonstrated encouraging efficacy in treating R/RMM patients who have previously received multiple therapies, including CD-38 monoclonal antibody therapy. However, immunotherapy also presents certain limitations, including the challenges associated with cell drug preparation, off-target effects, and toxic side effects. Therefore, it is imperative to continuously explore new MM antigen targets and further optimize the performance of immunotherapy drugs to maximize their efficacy while minimizing toxicity. Compared to traditional nanoparticles, exosomes offer advantages such as enhanced biocompatibility, stability, and reduced immunogenicity. This may provide a novel opportunity for targeted modification and loading of cytotoxic drugs in treating MM. However, the therapeutic efficacy, toxicity, in vivo distribution, stability, and mechanism of action should be further investigated in future studies.

In addition, synergistic effects between different immunotherapies and traditional chemotherapy agents may exist. With the increasing number of novel immunotherapy drugs entering clinical trials, it is imperative to evaluate the synergistic impact of diverse immunotherapies and conventional chemotherapeutic agents on anti-tumor immune responses while minimizing therapeutic toxicity. Furthermore, the efficacy and safety profiles of immunotherapy should be assessed across various stages of MM treatment and distinct prognostic groups to achieve a more rationalized and standardized drug regimen. It would be intriguing to investigate whether effective combinations can be achieved among different subsets of immune-binding drugs. In conclusion, the continuous advancement in new immunotherapy drugs has instilled fresh hope for achieving a cure for MM.

## Literature search

The PRISMA (Preferred Reporting Items for Systematic Reviews and Meta-Analyses) methodology was employed in this review. An electronic search was conducted using PubMed and the blood journal database up to 2023. The search terms included MM, cellular immunotherapy, checkpoint inhibitors, monoclonal antibodies, antibody-drug conjugates, bispecific antibodies, and exosomes. A total of 143 articles were retrieved from PubMed, along with five abstracts from the blood journal.
